# Rapid integrated clinical survey to determine prevalence and co-distribution patterns of lymphatic filariasis and onchocerciasis in a *Loa loa* co-endemic area: The Angolan experience

**DOI:** 10.1016/j.parepi.2017.05.001

**Published:** 2017-05-07

**Authors:** Miguel Brito, Rossely Paulo, Pedro Van-Dunem, António Martins, Thomas R. Unnasch, Robert J. Novak, Benjamin Jacob, Michelle C. Stanton, David H. Molyneux, Louise A. Kelly-Hope

**Affiliations:** a Centro de Investigacao em Saude de Angola/Health Research Centre of Angola, Caxito, Angola; b Lisbon School of Health Technology, Lisbon, Portugal; c Centre for Neglected Tropical Diseases, and Department of Parasitology, Liverpool School of Tropical Medicine, Liverpool, UK; d National Directorate of Public Health, Ministry of Health, Angola; e College of Public Health, Department of Global Health, University of South Florida, Florida, USA

**Keywords:** Sub-saharan Africa, Angola, Filariasis, Loiasis, *Loa loa*, Tropical eye worm, Severe adverse events, SAEs, Onchocerciasis, LF, Lymphatic filariasis, Elephantiasis, Mapping, Nodules, Hydrocoele, Lymphoedema, Ivermectin, RAPLOA, REMO, NTDs, Neglected tropical diseases

## Abstract

The Republic of Angola is a priority country for onchocerciasis and lymphatic filariasis (LF) elimination, however, the co-distribution of the filarial parasite *Loa loa* (loiasis) is a significant impediment, due to the risk of severe adverse events (SAEs) associated with ivermectin used in mass drug administration (MDA) campaigns. Angola has a high risk loiasis zone identified in Bengo Province where alternative interventions may need to be implemented; however, the presence and geographical overlap of the three filarial infections/diseases are not well defined. Therefore, this study conducted a rapid integrated filarial mapping survey based on readily identifiable clinical conditions of each disease in this risk zone to help determine prevalence and co-distribution patterns in a timely manner with limited resources. In total, 2007 individuals from 29 communities in five provincial municipalities were surveyed. Community prevalence estimates were determined by the rapid assessment procedure for loiasis (RAPLOA) and rapid epidemiological mapping of onchocerciasis (REMO) together with two questions on LF clinical manifestations (presence of lymphoedema, hydrocoele). Overall low levels of endemicity, with different overlapping distributions were found. Loiasis was found in 18 communities with a prevalence of 2.0% (31/1571), which contrasted to previous results defining the area as a high risk zone. Onchocerciasis prevalence was 5.3% (49/922) in eight communities, and LF prevalence was 0.4% for lymphoedema (8/2007) and 2.6% for hydrocoeles (20/761 males) in seven and 12 communities respectively. The clinical mapping survey method helped to highlight that all three filarial infections are present in this zone of Bengo Province. However, the significant difference in loiasis prevalence found between the past and this current survey suggests that further studies including serological and parasitological confirmation are required. This will help determine levels of infection and risk, understand the associations between clinical, serological and parasitological prevalence patterns, and better determine the most appropriate treatment strategies to reach onchocerciasis and LF elimination targets in the loiasis co-endemic areas. Our results also suggest that the utility of the earlier RAPLOA derived maps, based on surveys undertaken over a decade ago, are likely to be invalid given the extent of population movement and environmental change, particularly deforestation, and that fine scale micro-mapping is required to more precisely delineate the interventions required defined by these complex co-endemicities.

## 1 Introduction

The Republic of Angola is a priority country for onchocerciasis and lymphatic filariasis (LF) elimination in sub-Saharan Africa (World Health Organization, 2015a, World Health Organization, 2015b, World Health Organization, 2016a, World Health Organization, 2016b). These two human filarial infections are key neglected tropical diseases (NTDs) listed by the World Health Organization (WHO), and considered to be major public health problems, causing widespread disability and consequent poverty (World Health Organization, 2012a, World Health Organization, 2013a, World Health Organization, 2013b, World Health Organization, 2016c). Global elimination efforts primarily aim to interrupt transmission with preventive chemotherapy through mass drug administration (MDA) using ivermectin, albendazole and diethylcarbamazine (DEC) in different regimen combinations and to alleviate suffering through morbidity management and disability prevention (MMDP). While many countries in Africa have made steady progress in scaling up their national programmes, several countries, including Angola, are behind the targets if the WHO Roadmap is to be accomplished (Bockarie et al., 2013, Molyneux et al., 2016, World Health Organization, 2012a). In Central and West Africa, perhaps the most significant impediment for programmes is the co-distribution of the filarial parasite *L*. *loa*, due to the risk of severe adverse events (SAEs) including encephalopathy and death, which have been associated with ivermectin when given to individuals with high *L*. *loa* microfilariae (mf) loads in the blood (> 30,000 mf/ml) (Boussinesq, 2006, Gardon et al., 1997, Kelly-Hope et al., 2014, Twum-Danso, 2003).

The distribution of *L*. *loa* is restricted to the African equatorial rain forest and is transmitted by the tabanid flies *Chrysops* spp. (Kelly-Hope et al., 2017a). Typical clinical infections include Calabar swelling, pruritus and sub-conjuctival migration of the adult *L*. *loa* worm, which is an indication of- and referred to- as loiasis or Tropical Eye Worm (Boussinesq, 2006, World Health Organization, 2016c); however more recently a study has found excess mortality associated with high infection rates (Chesnais et al., 2016). The problem of SAEs associated with ivermectin was initially reported during MDA in onchocerciasis control programmes in Cameroon and later in the Democratic Republic of Congo (DRC), as a result of which, the African Programme for Onchocerciasis Control (APOC) (Cupp et al., 2011, Dadzie, 1997) developed the rapid assessment survey method for loiasis (RAPLOA) based on eye worm history (Addiss et al., 2003, Takougang et al., 2002, Wanji et al., 2005, Wanji et al., 2012). The extensive RAPLOA surveys undertaken have helped develop loiasis prevalence maps and predict areas at highest risk of SAEs (Takougang et al., 2002, Zouré et al., 2011). In parallel, advice to national onchocerciasis elimination programmes about how the risk of SAEs should be mitigated, and if they occur, be managed is based on the Guidelines established by the Mectizan Donation Programme Expert Committee (the Mectizan Expert Committee, MEC) (Addiss et al., 2003, Alleman et al., 2006, Colatrella, 2008, Ogoussan and Hopkins, 2011), now with technical expertise from the new Expanded Special Project for Elimination of Neglected Tropical Diseases (ESPEN) (Hopkins, 2016, World Health Organization, 2015a, World Health Organization, 2015b).

Angola has a *L*. *loa* high risk area in Bengo Province in the northwest region of the country with high prevalence estimates (> 40%) and associated risk of SAEs (Zouré et al., 2011). These risks were defined by RAPLOA surveys conducted in 2003, 2004 and 2008, and support historical studies on filariasis, which also found widespread onchocerciasis caused by *Onchocerca volvulus* and transmitted by the *Simulium* spp. vectors, but little evidence of the LF parasite *Wuchereria bancrofti* (Casaca, 1966a, Casaca, 1966b, Hawking, 1974). The current prevalence of LF in this area is not known, but is expected to be low based on the historical data and recent modelled map estimates developed by Cano et al. (2014), though these were based on a few data points. Baseline mapping is required to determine the way forward. However, it will be important to take into account the ongoing activities of the onchocerciasis programme that currently operates in some areas of Bengo Province.

Angola has seven high risk onchocerciasis areas being targeted with the annual community-directed treatment with ivermectin (CDTI) strategy, one of which overlaps with part of the loiasis high risk zone (World Health Organization, 2016a, World Health Organization, 2016b, World Health Organization, 2016c, World Health Organization, 2016d, Zouré et al., 2014). The CDTI areas were identified through the Rapid Epidemiological Mapping for Onchocerciasis (REMO) (Noma et al., 2002), which is based on community prevalence of skin nodules ≥ 20% in adults, as part of APOC's strategy to target and control blinding onchocerciasis (Ngoumou et al., 1994, Zouré et al., 2014). However, the recent change in strategy to expand and eliminate onchocerciasis, by treating low transmission areas (nodule prevalence < 20%) poses several challenges, which are being considered by ESPEN (Hopkins, 2015, Hopkins, 2016, Molyneux et al., 2014a, World Health Organization, 2015a, World Health Organization, 2015b). These challenges relate to the fact that hypo-endemic areas are geographically vast and difficult for health services to access, and not currently well-defined; ivermectin cannot be used in *L*. *loa* areas as the risk of SAEs outweighs the benefits to the community, and there is no recommended alternative safe strategy that is readily scalable in the *L*. *loa* co-endemic areas despite the development of the new 'test and not treat' (TNT) strategy (D'Ambrosio et al., 2015, Kamgno et al., 2016, Pion et al., 2016a).

The situation for implementing an alternative strategy for LF elimination in *L*. *loa* co-endemic areas is more straightforward, but not without challenges. While the WHO/Global Programme to Eliminate LF (GPELF), recommends the alternative strategy of twice a year albendazole together with the use of long-lasting/insecticide treated bed nets (LLIN/ITNs) (Kelly-Hope et al., 2013, World Health Organization, 2012b); only one or two countries out of the 10 *L*. *loa* co-endemic countries have started to implement this alternative strategy. The reasons for such delays are multi-faceted, but mainly related to political instability, poor infrastructure and difficult access to communities, (Molyneux et al., 2014a). A new practical approach for scaling up the alternative strategy may help countries to develop action plans, however they will need considerable funding and support at a national level (Kelly-Hope et al., 2017b). The other main challenge relates to measuring endemicity, which has largely been defined in Africa through mapping community prevalence of filarial antigen detected with the rapid diagnostic immuno-chromatographic test (ICT) card (BinaxNOW Filariasis), and now more recently the Filariasis Test Strip (FTS) (Weil et al., 2013, Weil and Ramzy, 2007). However, recent evidence indicates a cross-reactivity problem with the ICT card in high risk *L*. *loa* areas resulting in false positives and potentially an overestimation of LF prevalence (Bakajika et al., 2014, Pion et al., 2016b, Wanji et al., 2015, Wanji et al., 2016).

For the Angolan onchocerciasis and LF national programmes to implement MDA using ivermectin as a constituent drug, it is critical to understand the extent to which the three filarial infections overlap geographically. This will ensure that safe treatment strategies are implemented, and monitored for impact and potential SAEs. The large-scale RAPLOA and REMO surveys provide essential baseline information; however they were completed at different times, and on different and relatively large geographical scales. Micro-mapping and overlap-mapping are new approaches developed to delineate risk, define co-endemicity and target interventions which may be more useful in this loiasis high risk zone, which comprises both hyper- and hypo-onchocerciasis, and an unknown LF prevalence (Kelly-Hope et al., 2011, Kelly-Hope et al., 2014, Kelly-Hope et al., 2015, Okorie et al., 2013). Given that the programmes in Angola are behind targets for the achievement of elimination and have minimal funding support, the method to initially assess co-endemicity needs to be simple, rapid and relatively cost effective. Further for onchocerciasis, the use of skin snips and/or the new OV16 RDT (rapid diagnostic test) are currently not feasible or affordable at a large scale in this low-resource setting (Renneker et al., 2016). For LF, the problem of the cross-reactivity in *L*. *loa* endemic areas with the ICT card highlights that an alternative method is required, and in the absence of any new or alternative diagnostic tool, the presence of the main clinical symptoms of lymphoedema and hydrocoele may help to identify if LF is a public health problem.

This aim of this study was to support the national LF Programme and conduct an integrated filarial mapping survey to help determine the presence and co-distributions of onchocerciasis and LF in an area previously identified as one of high risk of *L*. *loa* SAEs. The study builds on the RAPLOA and REMO survey methods by adding LF clinical symptoms, and implements it on a fine geographical scale within this high risk zone, which also helps to construct a rapid integrated filarial ‘RAPLOA-REMO-LF’ clinical survey using a new micro-mapping approach.

## 2 Methods

### 2.1 Bengo Province

**Fig. 1:**
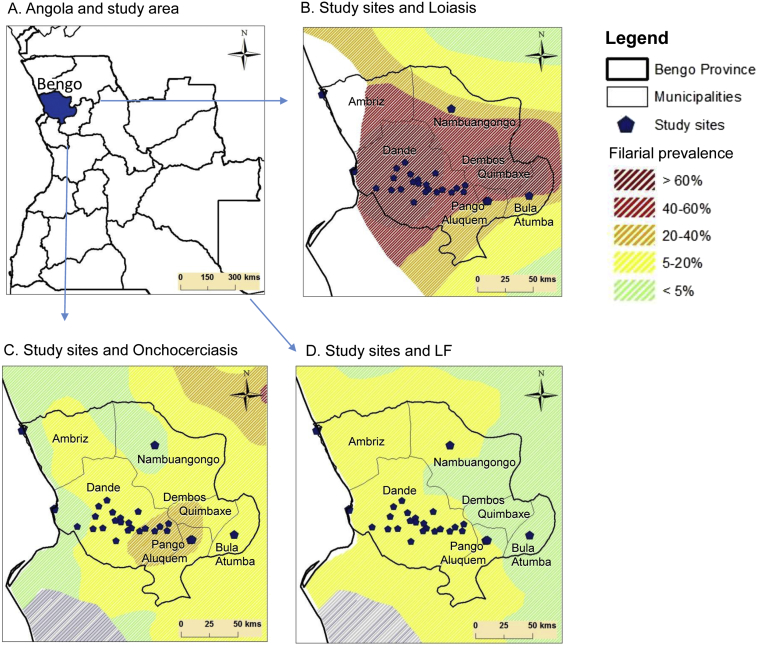
Study areas and location of study sites in relation to modelled filarial distributions. A. Angola and study area. B. Study sites and loiasis. C. Study sites and onchocerciasis. D. Study sites and LF.

The study was conducted in rural and semi-rural villages across six municipalities in the north-western province of Bengo, Angola where the loiasis endemicity was considered to be high as determined by a previous RAPLOA study, the onchocerciasis endemicity was moderate to low as determined by REMO, and the LF endemicity low as determined by recent published modelled maps, including historical records, which were imported into ArcGIS 10 (ESRI, Redlands CA) and digitized to created new maps with comparative prevalence/endemicity levels for the purpose of this study (Fig. 1A–D) (Cano et al., 2014, Zouré et al., 2011, Zouré et al., 2014). Bengo Province borders Luanda (south), Zaire (north), Uige (northeast), and Cuanza Norte (east) provinces, and has an area of 31,371 sq.km and a population of 351,579 with the majority of people living in rural areas with basic housing, and limited access to electricity, clean water or sanitation (Governo da República de Angola, 2014). Bengo Province has a tropical climate, with an average temperature of 25 °C (range 20°–29 °C) and a rainy season from October to April with peaks in November/December and March/April. It has large areas of rain forest as part of Kissama National Park and Kibinda Forest Reserve, as well as forest-savanna mosaic, savannah and woodland vegetation. Several large rivers run through the province including the Dande River, which flows rapidly from the higher eastern region to the western lowlands where it flows slowly through the capital, Caxito, into the Atlantic ocean.

### 2.2 Study sites

**Fig. 2:**
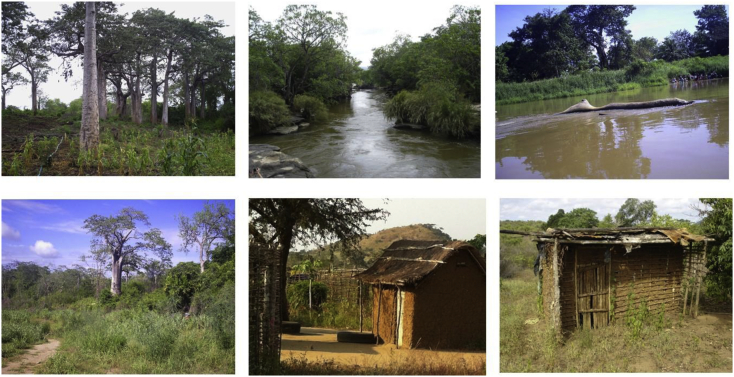
Photos of landscape and typical housing in the CISA study area.

The study was primarily focussed in the CISA area (Centro de Investigacao em Saude de Angola/Health Research Centre of Angola) of Dande Municipality, which comprises the three communes of Caxito, Mabubas and Ucua. The CISA includes the Dande Health Demographic Surveillance System (HDSS-Dande), which operates an ongoing population monitoring system across 69 geo-referenced neighbourhoods (here referred to as communities) comprising approximately 16,000 households, and a population of approximately 60,000 inhabitants in an area of 4700 sq.km (Costa et al., 2012). The population density is approximately 13 inhabitants per km^2^, and has both urban and rural characteristics, with 16 communities in the main town of Caxito. The landscape is primariy savanna with a forest gallery around the river banks and higher areas. The Dande, Lifune and Ucaua rivers run through the area and have permanent water flow, with many lakes surrounding lower parts of the Dande. Fig. 2 shows photos of the landscape, including rivers, vegetation and typical houses. In addition to the CISA area, additional communities from the adjacent municipalities of Ambriz, Nambuangongo, Dembos, Bula Atumba and Pango Aluquem were sampled in order to provide preliminary insights into the geographical extent and prevalence of filarial infections.

### 2.3 Mapping strategy and field logistics

To determine the prevalence and co-distribution of the three filarial diseases at a fine geographical scale across the CISA study area, a rapid integrated micro-mapping activity assessing the evidence of key clinical conditions was conducted in January and February 2014. One community was randomly selected from within a 15 km grid which was created in the geographical information system (GIS) software (ArcInfo 10, ESRI, Redland CA) to demarcate a 5 to 15 km distance between each, to ensure that prevalence was measured at regular spatial intervals across the entire study area and to incorporate a range of environmental characteristics.

In the adjacent five municipalities, only one or two communities were selected to provide insights into the endemicity in the surrounding CISA areas. Communities were included if they reported > 100 adult (> 15 years) inhabitants during the last DSS survey. In each community, all adults present at the time were invited to participate, and the first 100 individuals accepting the invitation were included in the survey. The mapping survey was implemented by the local CISA research team based in Caxito. In total five research assistants were trained in the detection of loiasis, onchocerciasis and LF clinical conditions using a short questionnaire and physical examination, in accordance with the rapid mapping assessment methods for loiais and onchocerciasis (described below). In each community, 100 individuals were targeted to be surveyed over a 2 to 3 week period, with the questionnaires administered in Portuguese.

### 2.4 Filarial clinical indicators

#### 2.4.1 *Loa loa*

To determine the prevalence of *L*. *loa* the rapid assessment procedure for loiaisis (RAPLOA) diagnostic survey method was used (Takougang et al., 2002, Zouré et al., 2011). The method was developed and validated by the WHO, reviewed by the Technical Consultative Committee of APOC and approved for loiasis mapping in Africa. RAPLOA is based on an individual's history of eye worm, with information obtained from a simple short non-invasive survey comprising three questions relating to past experience of eye worm, confirmed by a photograph of the *L*. *loa* adult worm in the white part of the eye, and with the duration of the most recent episode being between 1 and 7 days;

1. Have you ever experienced or noticed worms move along the white of your eye? (yes/no)
2. Have you ever had the condition in this picture? (yes/no)
3. How long (in days) did the worm stay before disappearing? (between 1 and 7; yes/no)

Loiasis was confirmed in an individual when the answers to the three questions were all positive ‘yes’. This is considered to be the restricted definition of eye worm or loiasis, and has been found to correlate with high *L*. *loa* mf rates and risk of SAEs (Addiss et al., 2003). An unrestricted definition of loiasis is considered to be a positive response to the first question only. In each community, individuals aged > 15 years and whom had resided in the area for at least 5 years were selected for assessment.

#### 2.4.2 Onchocerciasis

To determine the prevalence of onchocerciasis, the basis of the REMO method was used (Ngoumou et al., 1994, Noma et al., 2002). The standard REMO method was developed for APOC to delineate zones of endemicity, identify CDTI-priority areas and estimate the number of people to be treated. It samples a proportion of villages to determine the prevalence in the local area by feeling for sub-cutaneous worm nodules (caused by *Onchocerca volvulus* adult worms living under the skin) in 50 adult males, who are aged ≥ 20 years and have lived in the community for at least 10 years. If ≥ 20% of adults have nodules, the local area is considered to be a CDTI-priority area, and where nodule prevalence is < 20% then clinic-based treatment is applied. In this current study the REMO method was used. However, because the area was hypo-endemic both males and females were included.

#### 2.4.3 Lymphatic filariasis

To determine the presence of LF, an assessment of the main clinical conditions including limb lymphoedema (tissue swelling or thickening), and hydrocoele (scrotal swelling) was conducted. In Africa, the clinical conditions are caused by *Wuchereria bancrofti* adult worms lodging in the lymphatic system and disrupting the immune system and lymph carriage. Currently, there are no standard rapid mapping guidelines to detect LF clinical cases; however, new approaches using local health workers and simple mHealth tools have started to be used in the field (Stanton et al., 2016). For this study, the presence of lymphoedema and/or hydrocoele in individuals aged > 15 years was recorded and verified by a local medical officer. No severity of the condition was recorded.

### 2.5 Data analysis and mapping

All survey data were entered into Microsoft Excel 2010 by the CISA team, and IBM SPSS statistical version 21 software was used for analysis. Prevalence distributions by sex (male, female), and age class (15–19, 20–29, 30–29, 40–49, 50–59, ≥ 60) were summarised and statistical differences examined using the chi-square test (p-value significant < 0.05). The GPS locations of the communities and households within the CISA area were available from the DSS in Dande. The filarial prevalence in each CISA community was mapped using ArcGIS 10 (ESRI, Redland CA), and communities found to have more than five cases and multiple filarial diseases were further mapped by household to better understand the co-distribution at a micro-level.

### 2.6 Ethics, consent and patient referrals

The survey was approved by the Ethical Committees from the Ministry of Health of Angola (Conselho de ética do Ministério da Saúde de Angola) and the Liverpool School of Tropical Medicine Research. Written informed consent was obtained from each respondent and was orally explained if respondents were illiterate. A parent or guardian provided written informed consent on behalf of child participants. For those who refused to participate, no further questions were asked and no information was recorded. For those individuals who consented, their name, sex, age and years of residence in the community were recorded before proceeding with the questions about filarial clinical conditions. For individuals found to be positive for clinical conditions, information on risk factors and prevention were provided, and they were referred to the local health services, which had previously been informed of the survey's activities. As part of the DSS in Dande, an established communication system is already in place between the CISA team and all community clinics, therefore good support and follow-up for the patients could be provided.

## 3 Results

### 3.1 Field work

The study was conducted in January and February 2014. In total, 29 community study sites were sampled across the six municipalities with the majority from the CISA area in Dande Municipality (Table 1). All study sites were within endemic areas of loiasis, onchocerciasis and LF as defined by the modelled maps with a range of prevalence distributions as shown in the digitized maps in Fig. 1A–D. For loiasis, all sites were in pre-defined high risk ‘hyper-endemic’ areas (> 40%), for onchocerciasis the majority of sites were in pre-defined low risk ‘hypo-endemic’ areas (< 20%) with approximately one third in CDTI priority areas (≥ 20%), and for LF, all sites were in very low (< 5%) or low (5–20%) ‘hypo-endemic’ areas as defined by the modelled mf map.

**Table 1:** Summary of study sites, population and individual samples.

Municipality	Population^a^	N of sampled villages	N examined	N males	N females	Average age (years)
Dande	217,929	23	1.380	495	885	41.00
Ambriz	21,806	2	191	51	140	39.18
Pango-Aluquem	6,571	1	99	54	45	31.00
Dembos-Quimbaxe	28,202	1	117	56	61	26.00
Bula Atumba	16,047	1	116	60	56	36.39
Nambuangongo	61,024	1	104	45	59	34.67
Total	351,579	29	2.007	761	1246	40.09

a Total population recorded during the National Census 2014.

Approximately half of the sites (n = 13; 48.1%) had lower than the expected number of adults meeting the inclusion criteria, which is likely due to population movements associated with long-term civil conflict, seasonal (agricultural) employment, charcoal activity and rural-to-urban immigration trends, which is now common in this area after the war. This limited the analysis as it was not possible to include the necessary sample size of 100 individuals or assess the prevalence in those individuals, who are most often men, as they were not present, apparently working outside the village.

**Fig. 3:**
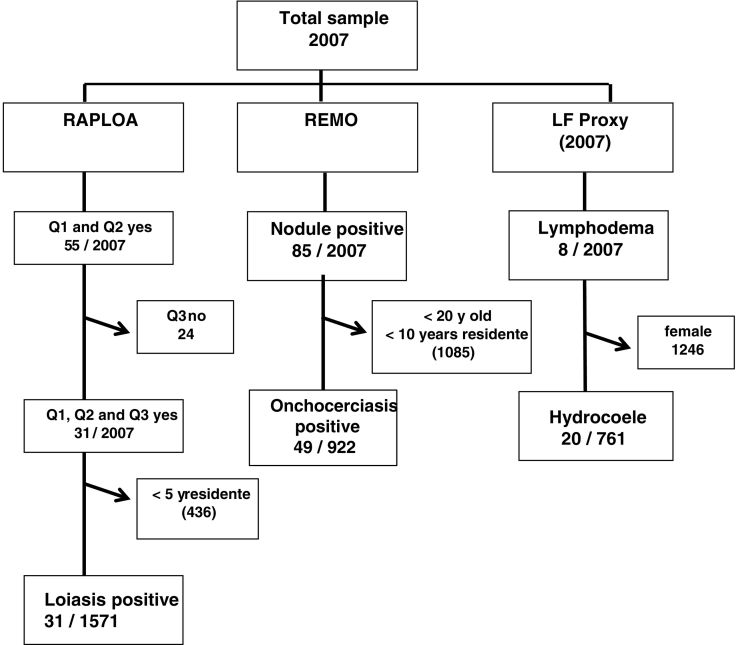
Sampling and analysis framework.

In total, 2007 individuals were surveyed, with numbers ranging from nine to 149 in each community study site. There were 761 males (37.9%) and 1246 females (62.1%), with average age of 40 years, ranging from 15 to 94 years based on 1558 individuals reporting their age. Fig. 3 presents the sampling and analysis framework for each disease with details on the numbers, prevalence rates by age, sex and community study site described below and outlined in Tables 2–6.

**Table 2:** Summary of loiasis distribution measured by RAPLOA methods.

Municipality	Village	N examined	N included RAPLOA^a^	Positives loiasis	% Positive loiasis
Dande		1380	1010	18	1.8
		200	144	1	0.7
	Açucareira Sede	99	68	1	1.5
	Sassa Povoação	101	76	0	0.0
		670	501	12	2.4
	Boa Esperança 2	100	89	2	2.2
	Bunba	41	26	2	7.7
	Honga Hungo	46	33	1	3.0
	Icau Centro	24	22	1	4.5
	Jungo	99	65	2	3.1
	Kilometro 29	27	20	2	10.0
	Mabubas	125	95	1	1.1
	Mazaza	25	18	0	0.0
	Muceque Teba	34	18	0	0.0
	Muculo	52	44	1	2.3
	Quilengues	32	30	0	0.0
	Santa Ambuleia	65	41	0	0.0
		62	41	2	4.8
	Caprédio	9	4	0	0.0
	Lifune Napasso Kicabo	53	37	2	5.4
		448	324	3	0.9
	Catuta	36	31	0	0.0
	Cherú	48	37	1	2.7
	Coragem	100	67	1	1.5
	Kacamba	25	11	0	0.0
	Mussenga	98	71	0	0.0
	Três Casas	92	74	1	1.4
	Vida e Sacrificio	49	33	0	0.0
Ambriz		191	185	4	2.2
	Capulo	42	42	1	2.4
	Tabi	149	143	3	2.1
Pango-Aluquem					
	Cazuangono	99	87	2	2.3
Dembos-Quimbaxe					
	Coqueiros	117	87	0	0
Bula Atumba					
	Ibundo	116	106	2	1.9
Nambuangongo					
	Muxaluando	104	96	5	5.2
Total Bengo	–	2007	1571	31	2.0%

a Persons older than 15 years old and that have been resident in the village for at least 5 years.

### 3.2 RAPLOA survey

For loiasis, 1571 individuals were included in the community analysis based on the RAPLOA inclusion criteria. In total, 346 individuals (22.0%) stated that had experienced a worm moving in their eye; of those 55 individuals (16% of 346; 3.5% of 1571) confirmed the worm with the picture shown by the interviewer, with 31 individuals (56% of 55; 2% of 1571) confirming the most recent episode being between 1 and 7 days (Table 2, Fig. 3). There were no significant differences between males and females in the unrestricted and restricted eye worm definitions (Table 5). Of the 29 communities surveyed, a total of 18 communities were found to have loiasis positive individuals based on the restricted definition.

**Fig. 4:**
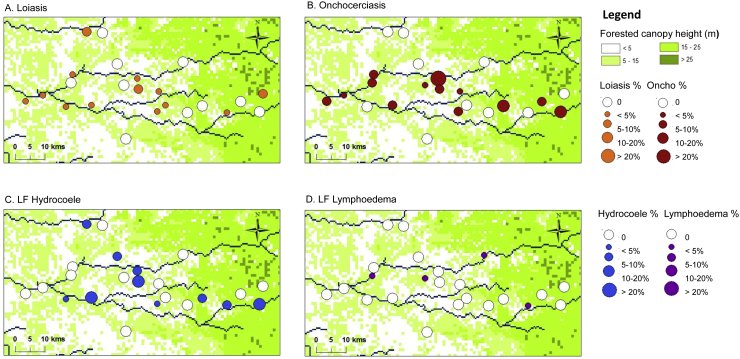
Filarial prevalence distributions in CISA communities. A. Loiasis. B. Onchocerciasis. C. LF Hydrocoele. D. LF Lymphoedema.

Overall, prevalence ranged from 0% to 10%, and the distribution in the CISA areas only is shown in Fig. 4A, which highlights that positive communities are located throughout the area with no obvious geographical pattern or clustering. When examining prevalence by age and sex (based on 1558 individuals who provided information), overall no significant differences between males and females or by age class were found (Table 4). Community-level age and sex analysis was not possible due to the low numbers, with most communities reporting one or two loiasis cases, and only Tabi (Ambriz Municipality) and Muxaluando (Nambuangongo Municipality) reporting three to five positive cases including both sexes and a range of age classes.

### 3.3 REMO survey

For onchocerciasis, there were 922 individuals from eight communities who were included in the analysis based on the REMO inclusion criteria, with 49 individuals (5.3%) found to have palpable nodules (Table 3; Fig. 3). Of the 29 communities surveyed, initially 18 communities found positive individuals, however only eight communities met the required minimum numbers of 50 male adults. For the purpose of this study in a hypo-endemic area, all data were included in the analysis and mapped. Overall, the prevalence ranged from 4.8% to 7.6% in the eight REMO communities, and from 2.9% and 42.9% in the other communities with fewer individuals surveyed. The distribution of all communities in the CISA area is shown in Fig. 4B, which highlights that the positive communities are located throughout the study area with no specific geographical pattern. When examining prevalence by age, overall there was an increasing prevalence by age, which was found to be significant overall, and in the female sub-group (Table 6). Community-level age and sex analysis was not possible due to the low numbers of only three to seven positive cases found in each community.

**Table 3:** Summary of onchocerciasis nodules distribution measured by REMO methods.

Municipality commune	Village	N examined	N included REMO^a^	Positives nodules	% Positive nodules
Dande		1380	486	26	5.3
Caxito		200	97	6	6.2
	Açucareira Sede	99	34	1	2.9
	Sassa Povoação	101	63	5	7.9
		670	261	15	5.7
	Boa Esperança 2	100	66	5	7.6
	Bunba	41	18	0	0.0
	Honga Hungo	46	7	3	42.9
	Icau Centro	24	16	1	6.3
	Jungo	99	23	1	4.3
	Kilometro 29	27	10	1	10.0
	Mabubas	125	55	3	5.5
	Mazaza	25	7	0	0.0
	Muceque Teba	34	8	0	0.0
	Muculo	52	18	0	0.0
	Quilengues	32	7	0	0.0
	Santa Ambuleia	65	26	1	3.8
		62	24	0	0.0
	Caprédio	9	1	0	0.0
	Lifune Napasso Kicabo	53	23	0	0.0
		448	104	5	4.8
	Catuta	36	21	2	9.5
	Cherú	48	25	0	0.0
	Coragem	100	19	0	0.0
	Kacamba	25	7	1	14.3
	Mussenga	98	8	1	12.5
	Três Casas	92	17	1	5.9
	Vida e Sacrificio	49	7	0	0.0
Ambriz		191	163	7	4.3
	Capulo	42	33	0	0.0
	Tabi	149	130	7	5.4
Pango-Aluquem					
	Cazuangono	99	63	3	4.8
Dembos- quimbaxe					
	Coqueiros	117	50	3	6.0
Bula Atumba					
	Ibundo	116	86	5	5.8
Nambuangongo					
	Muxaluando	104	74	5	6.8
Total Bengo	–	2007	922	49	5.3%

a Persons older than 20 years old and that have been resident in the village for at least 10 years.

### 3.4 Lymphatic filariasis clinical signs

For LF, all 2007 individuals were eligible for inclusion in this study. In total, eight individuals (0.4%) were found to have leg lymphoedema and 20 men were found to have hydrocoele (2.6%) (Table 4; Fig. 3). Of the 29 communities surveyed, a total of seven communities reported lymphoedema cases, 12 communities reported hydrocoele cases and three communities reported both clinical conditions, however, different individuals were affected. For lymphoedema, no significant differences by age and sex were found; however for hydrocoele, there was an increasing prevalence by age class with significant differences found (P-value < 0.001). The highest hydrocoele prevalence rates were reported among men aged over 50 years, which ranged from 3.4% to 8.5% (Table 6). The distribution of LF clinical cases for the CISA area are shown in Fig 4C and D, and highlight that there is no specific geographical pattern for either condition and a very low prevalence of lymphoedema in the area.

**Table 4:** Summary of clinical lymphatic filariasis distribution measured by the presence of lymphoedema and hydrocoele.

Municipality	Village	N examined	Positives lymphoedema	% Positive lymphoedema	N male	Positives hydrocoele	% Positive hydrocoele
Dande							
Caxito							
	Açucareira Sede	99	0	0.0	24	0	0.0
	Sassa Povoação	101	1	1.0	32	0	0.0
	Boa Esperança 2	100	0	0.0	30	0	0.0
	Bunba	41	0	0.0	17	0	0.0
	Honga Hungo	46	0	0.0	15	1	6.7
	Icau Centro	24	0	0.0	8	1	12.5
	Jungo	99	0	0.0	33	0	0.0
	Kilometro 29	27	0	0.0	10	2	20.0
	Mabubas	125	0	0.0	37	0	0.0
	Mazaza	25	1	4.0	15	0	0.0
	Muceque Teba	34	0	0.0	21	2	9.5
	Muculo	52	0	0.0	24	1	4.2
	Quilengues	32	0	0.0	16	0	0.0
	Santa Ambuleia	65	2	3.1	32	0	0.0
	Caprédio	9	0	0.0	4	0	0.0
	Lifune Napasso Kicabo	53	0	0.0	14	1	7.1
	Catuta	36	0	0.0	7	0	0.0
	Cherú	48	1	2.1	16	1	6.3
	Coragem	100	0	0.0	29	0	0.0
	Kacamba	25	0	0.0	14	2	14.3
	Mussenga	98	0	0.0	43	4	9.3
	Três Casas	92	0	0.0	34	1	2.9
	Vida e Sacrificio	49	0	0.0	20	0	0.0
Total							
Ambriz							
	Capulo	42	0	0.0	5	0	0.0
	Tabi	149	1	0.7	46	2	4.3
Total		191	1	0.5	51	2	3.9
Pango-Aluquem							
	Cazuangono	99	0	0.8	0	0	0.0
Dembos- quimbaxe							
	Coqueiros	117	1	0.9	56	0	0.0
Bula Atumba							
	Ibundo	116	0	0.0	60	0	0.0
Nambuangongo							
	Muxaluando	104	1	1.0	45	2	4.4
Total	–	2007	8	0.4	761	20	2.6

**Table 5:** Comparison of loiasis prevalence based on restricted and unrestricted RAPLOA definitions.

	N examined	N included in RAPLOA^a^	N Positives yes to 1 question	% Positives yes to 1 question	N Positives yes to 2 questions	% Positives yes to 2 questions	N Positives yes to 3 questions	% Positives yes to 3 questions
Total	2007	1571	346	22.0	55	3.5	31	2.0
Male	761	592	117	19.7	17	2.9	15	2.0
Female	1246	979	229	23.4	38	3.9	25	2.0

a Persons older than 15 years old and that have been resident in the village for at least 5 years.

**Table 6:** Prevalence of loiasis, onchocerciasis and lymphatic filariasis by sex and age class.

Prevalence									
		*Loiasis*		Onchocerciasis		Lymphatic filariasis			
						Lymphoedema		Hydrocoele	
	Age class	N	%	N	%	N	%	N	%
Total	15 to 19	167	1.8	–	–	254	0.8	–	–
	20 to 29	312	1.3	214	1.4	439	0.2	–	–
	30 to 39	275	2.9	199	3.5	356	0.6	–	–
	40 to 49	242	2.1	149	5.4	292	0.0	–	–
	50 to 59	269	1.5	164	7.9	318	0.3	–	–
	> 60	293	2.4	196	9.2	332	0.6	–	–
	Chi-square	ns		p < 0.001		Ns		–	–
Male	15 to 19	80	2.5	–	–	116	0.0	116	0.0
	20 to 29	110	0.0	86	1.2	157	0.6	157	0.6
	30 to 39	92	3.3	65	6.2	118	0.8	118	0.8
	40 to 49	86	2.3	51	3.9	108	0.0	108	1.9
	50 to 59	95	1.1	51	7.8	116	0.0	116	3.4
	> 60	125	1.6	87	6.9	142	0.7	142	8.5
	Chi-square	ns		Ns		Ns		p < 0.001	
Female	15 to 19	87	1.1	–	–	138	1.4	–	–
	20 to 29	202	2.0	128	1.6	282	0.0	–	–
	30 to 39	183	2.7	134	2.2	238	0.4	–	–
	40 to 49	156	1.9	98	6.1	184	0.0	–	–
	50 to 59	174	1.7	113	8.0	202	0.5	–	–
	> 60	168	3.0	109	11.0	190	0.5	–	–
	Chi-square	ns		p < 0.001		Ns		–	–

### 3.5 Micro-mapping co-distributions

Overall, there was no distinct geographical pattern of the presence or absence of the different filarial diseases in each of the CISA study communities. Four communities were found to co-endemic for all three filarial diseases, and four communities had none. The presence of loiasis cases alone was found in two villages, onchocerciasis cases alone in one community, and LF cases alone in two communities. The presence of loiasis and onchocerciasis cases was found in four communities, and loiasis and LF cases in two communities, while the presence of onchocerciasis and LF cases was found in four communities.

**Fig. 5:**
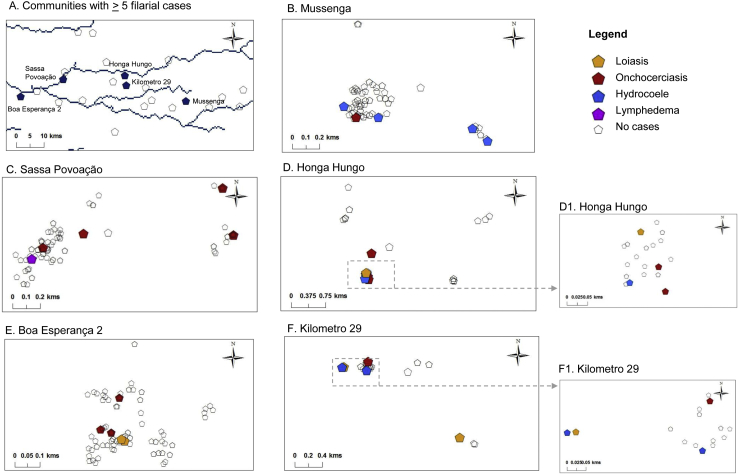
Micro-mapping filarial cases in high risk communities.

The communities of Boa Esperança 2, Honga Hungo, Kilometro 29, Mussenga, Sassa Povoação were found to have ≥ 5 filarial cases and were located across the study area (Fig. 5A). The results from the micro-mapping are presented in Fig. 5B–F, and highlight the ‘within-community’ distribution of each filarial disease. Only one individual from one household in Boa Esperança 2 community was found to have clinical conditions related to two diseases (loiasis and onchocerciasis) while all other communities reported the presence of cases from different households, indicating the absence of co-infection.

## 4 Discussion

This is the first survey in Angola to determine the presence of three filarial infections using rapid field methods based on key disease specific clinical conditions. The approach builds on the well-established RAPLOA and REMO survey methods, which have been used extensively across Central and West Africa to define prevalence distributions and direct treatment strategies (Noma et al., 2002, Takougang et al., 2002, Zouré et al., 2014). To-date, only one integrated RAPLOA-REMO survey in neighbouring DRC has been reported, which found significant differences in co-endemicity across the country (Tekle et al., 2011). Bordering Angola, the Bas Congo region in DRC had reported SAEs (World Health Organization, 2004a, World Health Organization, 2004b), and the availability of both loiasis and onchocerciasis data, enabled simple maps and models to be developed to highlight the specific high risk areas (Kelly-Hope et al., 2015, 2014). This is particularly important for Angola, as the current study in Bengo Province was conducted in a previously defined high risk area (Zouré et al., 2011), where all three diseases were found to be present.

The rapid integrated filarial ‘RAPLOA-REMO-LF’ clinical survey method used here in Angola also included a new micro-mapping approach to determine prevalence distributions at a fine geographical scale. Study communities were mapped on a scale of ~ 5-15 km apart, which helped to highlight the wide variability in prevalence of all three infections in the relatively small CISA area (Costa et al., 2012). However, due to some small community sample sizes, and a highly mobile population with some potentially ‘at risk individuals’ absent from the study, accurate estimates of risk were compromised. This fact highlights how risk and population dynamics can change over time, and that there is a need for up-to-date information and assessments before treatment strategies are implemented. Notwithstanding, these limitations the overall prevalence was low (defined as meso-to-hypo endemic), with no obvious spatial patterns found, the communities with a higher risk of one or more diseases were readily identifiable. This has provided important preliminary information to the national programmes as a ‘first step’ in understanding the local filarial epidemiology, and will help to investigate community risk in more detail as a ‘second step’ by assessing serological and parasitological prevalence rates, and other potential risk factors including the main vectors of loiasis (*Chrysops* spp.) and onchocerciasis (*Simulium* spp.). It will also help to determine if alternative intervention strategies are required (Kelly-Hope et al., 2015, Kelly-Hope et al., 2017a, Kelly-Hope et al., 2017b).

For loiasis, a significant difference was found between the current survey data with the RAPLOA modelled maps from 2010 (Zouré et al., 2011). The reason for this significant difference is unclear, but may be related to the timing and spatial resolution of data collected as previous surveys were only conducted in a few villages between 2003 and 2008 (Zouré et al., 2011). It may also be related to levels of deforestation, tree cover change, recent seasonal migration patterns as many people were not available for the survey, and/or urbanisation changes in the area (Costa et al., 2012, Governo da República de Angola, 2014, Hansen et al., 2013, World Resources Institute, 2017). Further investigations are needed to better determine *L*. *loa* prevalence, as it may be underestimated. If the *L*. *loa* risk is found to be meso- endemic, then the risk of SAEs is also likely to be higher. However, the proportion of individuals with high *L*. *loa* mf loads (≥ 30,000 ml) in such meso-endemic areas is unknown as only a few studies have been conducted, and primarily focussed on the relationship between RAPLOA and mf rates in high transmission areas (Addiss et al., 2003, Boussinesq et al., 2001, Schlüter et al., 2016, Takougang et al., 2002, Wanji et al., 2012). Therefore, new investigations in these meso-‘intermediate/transition’ zones are required, potentially using alternative measurement tools such as the CellScope Loa through the test and not treat (TNT) strategy (D'Ambrosio et al., 2015), which may be better able to address these complex risk areas, and help develop ‘intermediate transmission’ models to better predict risk of SAEs.

For onchocerciasis and LF, there was more correlation between the current survey results and modelled levels of endemicity. However, it is important to note that higher onchocerciasis prevalence was found in drug naïve communities outside the defined CDTI area, and coincided with communities with a high prevalence of loiasis. A better understanding of the risks and benefits of extending the current CDTI boundaries or whether alternative strategies including doxycycline (Molyneux et al., 2003), and/or vector control for both *Simulium* spp. and *Chrysops* spp. are required (Kelly-Hope et al., 2017a). An extensive review of *Chrysops* spp. suggests that various forms of vector control (tiny targets as used in tsetse control) or new repellent approaches to deter biting of day biting vectors [emanators] may reduce risk and thus *L*. *loa* mf loads, which could be a novel intervention in low onchocerciasis transmission areas (Kelly-Hope et al., 2015, Kelly-Hope et al., 2017a). Such hypo-endemic onchocerciasis areas are now priority for ESPEN (World Health Organization, 2015a, World Health Organization, 2015b), who could provide further strategic, operational and technical support for the implementation and systematic monitoring of safe and effective strategies.

For LF, the few clinical cases verified by medical officers helped to confirm that they were not inguinal hernias. There may be more and/or missed hydrocoele cases in the community given the mobility of the population and social sensitivities of exposing legs and genitalia. However, it is likely that the well-monitored DSS operating in this region would have identified the magnitude of the problem already. While the hydrocele rates in low endemic areas may not be a reliable predictor of prevalence compared with highly endemic communities (Eigege et al., 2003, Gyapong et al., 1998), the data indicate that transmission is low, which is in accordance with historical data (Casaca, 1966a, Casaca, 1966b). A random sample of night bloods to detect microfilaraemia may have helped to confirm the low LF prevalence, and could be included in the more detailed ‘second step’ serological assessments in the future. Nonetheless, this initial clinical survey, suggests that LF transmission may be readily interrupted with the WHO recommended alternative strategy of albendazole twice yearly plus vector control (Kelly-Hope et al., 2017b, World Health Organization, 2012b).

Further for LF, collaborative links with the national malaria control programme will be essential to help increase bed net coverage, which is very low with only around one third of households owning an ITN (Cosep Consultoria Consaúde and ICF International, 2011). Morbidity management and disability prevention may be readily addressed through home-based lymphoedema care, and surgery for the few men identified with hydrocoele (World Health Organization, 2013a, World Health Organization, 2013b). This epidemiological pattern of low LF prevalence in *L*. *loa* endemic area has been found elsewhere (Bakajika et al., 2014, Bregani et al., 2007, Tekle et al., 2011), and supports the idea that elimination may be more easily achieved in these co-endemic areas than previously thought. This LF-loiasis pattern also supports the idea of competitive exclusion of filarial parasites in Africa (Molyneux et al., 2014b), and the lack of co-infection at individual and household level found in this study supports this, and similar to another integrated mapping study conducted in Nigeria (Brant, 2014).

The filarial endemicity found in Bengo Province is potentially complex. Therefore, a better understanding of the extent and intensity of serological and parasitological co-infections is essential, and how risk and populations may have changed since the original loiasis mapping is required for the scale-up of safe and effective treatment strategies (Molyneux et al., 2014a). It is feasible that the rapid integrated clinical survey method presented here could be conducted across a larger geographical region in selected ‘data naïve’ co-endemic areas as an initial risk mapping model. This will highlight the range of co-endemic patterns in the different regions of the country, and provide a broader perspective of the potential resources, specific investigations and technical expertise that may be needed. This is important as the Angolan LF and onchocerciasis elimination programmes will face several challenges in implementing and monitoring the impact of several intervention strategies across the country (Molyneux et al., 2014a). It will require significant collaboration, and human and financial support from international partners and stakeholders over the next 5 years in order to accelerate activities to meet national targets and global goals (World Health Organization, 2012a, World Health Organization, 2015a, World Health Organization, 2015b).

## Competing interests

The authors declare that they have no competing interest.
